# Anthocyanins of Coloured Wheat Genotypes in Specific Response to SalStress

**DOI:** 10.3390/molecules23071518

**Published:** 2018-06-23

**Authors:** Sonia Mbarki, Oksana Sytar, Marek Zivcak, Chedly Abdelly, Artemio Cerda, Marian Brestic

**Affiliations:** 1 National Research Institute of Rural Engineering, Water and Forests (INRGREF), BP 10, Aryanah 2080, Tunisia; mbarkisonia14@gmail.com; 2 Laboratory of Plant Extremophiles, Biotechnology Center at the Technopark of Borj-Cedria Tunisia, BP 901, Hammam Lif 2050, Tunisia; chedly.abdelly@cbbc.rnrt.tn; 3 Department of Plant Biology, Institute of Biology, Kiev National University of Taras Shevchenko, Volodymyrska St, 64, 02000 Kyiv, Ukraine; oksana.sytar@gmail.com; 4 Department of Plant Physiology, Slovak University of Agriculture, Nitra, Tr. A. Hlinku 2, 949 01 Nitra, Slovak Republic; marek.zivcak@uniag.sk (M.Z.); marian.brestic@uniag.sk (M.B.); 5 Departament of Geografy, University of València, Blasco Ibàñez, 28, 46010 Valencia, Spain; artemio.cerda@uv.es

**Keywords:** salinity, anthocyanins, proline, MDA, flavonol, wheat

## Abstract

The present study investigated the effect of salt stress on the development of adaptive responses and growth parameters of different coloured wheat genotypes. The different coloured wheat genotypes have revealed variation in the anthocyanin content, which may affect the development of adaptive responses under increasing salinity stress. In the early stage of treatment with salt at a lower NaCl concentration (100 mM), anthocyanins and proline accumulate, which shows rapid development of the stress reaction. A dose-dependent increase in flavonol content was observed for wheat genotypes with more intense purple-blue pigmentation after treatment with 150 mM and 200 mM NaCl. The content of Na^+^ and K^+^ obtained at different levels of salinity based on dry weight (DW) was more than 3 times greater than the control, with a significant increase of both ions under salt stress. Overall, our results demonstrated that coloured wheat genotypes with high anthocyanin content are able to maintain significantly higher dry matter production after salt stress treatment.

## 1. Introduction

Land degradation has become currently a strong issue at a global level as its spatial distribution varies from 1 billion ha to over 6 billion ha [1]. Climate change, high salt levels in soils and irrigation waters are major environmental concerns, and a problem for agriculture in many arid and semi-arid regions [2]. Approximately 15% of the cultivated land has an excess of salt [3], and large quantities of water are of very poor quality. Globally, no less than 10 million hectares of agricultural land are abandoned annually because of salinity. Salt-induced land degradation is a very important issue affecting the status of food productivity worldwide [2,3,4].

Salinity can increase the toxic levels of some ions and cause water stress and malnutrition in plants. The difference in salt tolerance may be connected with plant ontogenetic stages and plant species. It is usually visible in the reduction of biomass and yield or in decreasing survival rates [5,6]. In plants, osmotic stress and ionic toxicity are two stresses that follow salinity and can be destructive under prolonged treatment [5]. Plants respond to these variations in soil salinity by provoking resistance mechanisms [7,8]. Among known mechanisms, osmotic adjustment plays a major role in plant tolerance to stress [9]. A high level of Na^+^ compromises carbon fixation, which supports an over-reduction of light-harvesting complexes, reducing photosynthesis and the production of reactive oxygen (ROS) [10]. This process generates a disturbance in ionic homeostasis and/or nutritional imbalance [11].

Under stress conditions, secondary metabolite accumulation often occurs in plants [12]. Secondary metabolites may participate as signal molecules and some specific elicitors. Ionic and osmotic stresses, which are part of salt stress in plants, stimulate the accumulation or decrease of specific secondary metabolites in plants [13]. Anthocyanins are increased during the salt stress response [14]. At the same time, salt stress is able to decrease the level of anthocyanins in salt-sensitive plant species [14]. Flavonoids are one of the largest groups of plant phenolics that participate in plant defence [15] via an effective strategy against ROS [16]. Under salt stress, *Azospirillum brasilense* showed higher secretion of nod-gene-inducing flavonoid species and improved root branching in the seedling roots of bean plants [17]. Other salt-sensitive crops like potato, eggplant, pepper, cabbage, lettuce, rise and maize shows their sensitivity to hyperosmotic stresses by the rise of phenolics, as well [18,19,20,21]. Under drought and salinity stresses, wheat plants raise antioxidant defence mechanisms under abiotic stresses to alleviate oxidative damage [22,23].

It was suggested that accession-dependent capacity to stimulate salt-stress response antioxidative mechanisms may affect a corresponding changeability for the growth sustainability of some plants. The high leaf antioxidant activity and total phenolic content have been estimated in the halophyte *Cakile maritima* leaves [24]. The wheat genotypes demonstrated a wide range of responses to high salinity stress during the growth stages [25]. The effect of salt stress on wheat plants is one of the most serious problems in arid and semi-arid regions, which reduces both the quantity and quality of the production of this cereal [26].

The response to the salt stress of plant species depends on the plant species and varieties, salt concentration, growing conditions and stage of development of the plant. The identification of salt-tolerant varieties can positively develop the production of areas at risk or irrigated with salinized water and would be of obvious interest in helping to improve varieties.

Therefore, the purpose of this work was to study the effect of salt stress on the development of adaptive responses and growth parameters of different wheat cultivars (coloured and not coloured) to estimate parameters that can help to choose salt-tolerant wheat cultivars.

## 2. Results

### 2.1. SFR, ANTH, FLAV and MFI

**Figure 1:**
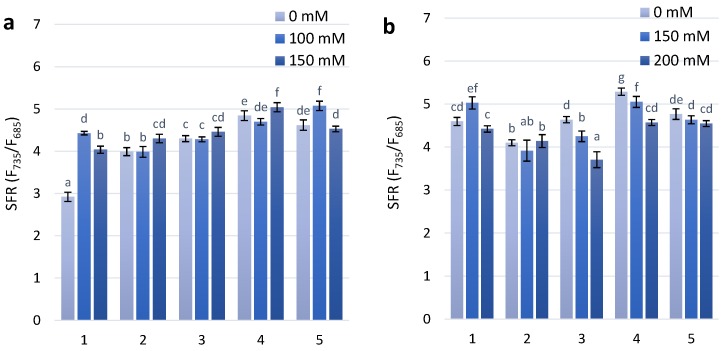
Values of SFR (simple fluorescence ratio) in the leaves of wheat plant genotypes exposed to salt stress during 30 days after seedlings (**a**)—first stage of treatment; (**b**)—second stage of treatment. The numbers indicate individual cultivars of wheat as follow: 1—Citrus yellow, 2—KM 53-14 Blue, 3—KM 178-14 purple, 4—Skorpion Blue aleurone, 5—PS Karkulka purple. The columns represent the mean values ± S.E. for six replicates. Statistically significant differences among treatments at each time are indicated by different small letters (Duncan test, *p* < 0.05).

The results of the presented experimental work showed that the SFR values at the first stage of treatment with a concentration of 100 mM increased in two genotypes, Citrus yellow and PS Karkulka. Under 150 mM NaCl, increasing SFR values have been observed in the non-coloured genotypes (Citrus yellow) at the first stage of treatment and continue in the second stage of treatment. For the two coloured genotypes, KM 178-14 purple and Skorpion Blue aleurone under the first stage of treatment with 100 mM and 150 mM NaCl showed no significant changes compared to the control. At the second stage of treatment, with increasing salt concentration (150 mM and 200 mM NaCl), the SFR value decreased (Figure 1).

**Figure 2:**
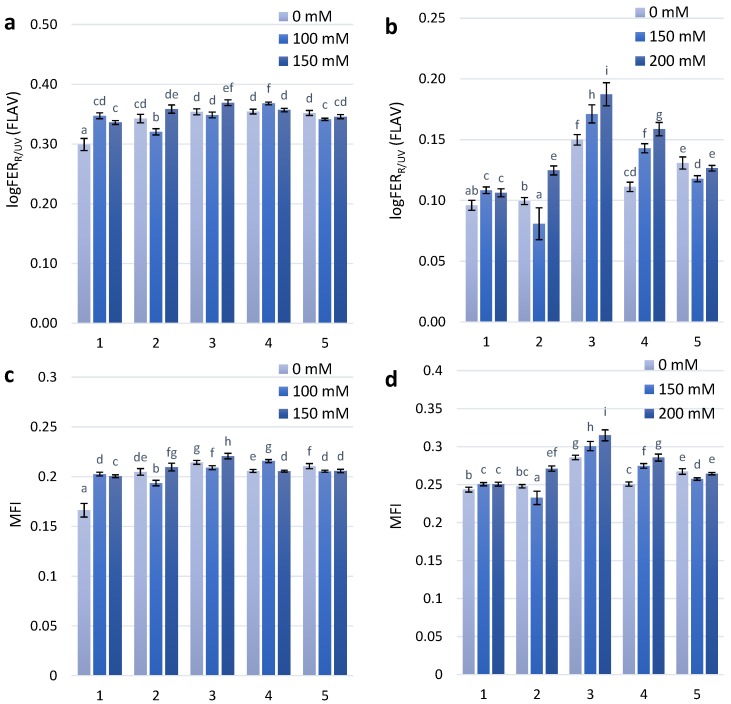
Flavonols accumulation (logFERR/UV-FLAV) in the leaves of investigated wheat plant genotypes exposed to salt stress during 30 days after seedlings (**a**)—first stage of treatment; (**b**)—second stage of treatment. MFI parameter in the leaves of investigated wheat plant genotypes exposed to salt stress during 30 days after seedlings (**c**)—first stage of treatment; (**d**)—second stage of treatment.The numbers shown individual cultivars of wheat: 1—Citrus yellow, 2—KM 53-14 Blue, 3—KM 178-14 purple, 4—Skorpion Blue aleurone, 5—PS Karkulka purple The columns represent the mean values ± S.E. for six replicates. Statistically significant differences among treatments at each time are indicated by different small letters (Duncam test, *p* < 0.05).

Flavonol accumulation, which is also part of the adaptive reaction to salt stress, was observed slightly after the first stage of treatment with NaCl concentrations of 100 mM and 150 mM. At the second stage of treatment with 150 mM and 200 mM NaCl, there was a significant difference in the flavonol reaction between the non-coloured and coloured wheat genotypes (Figure 2A). A dose-dependent increase in flavonol content was observed for the KM 178-14 purple and Skorpion Blue aleurone wheat genotypes after treatment with 150 mM and 200 mM NaCl, respectively (Figure 2B).

The modified flavonoid index (MFI), which takes into consideration the accumulation of both flavonols and anthocyanins, is a better estimate of flavonoids than FLAV. The results of statistical analyses using MFI are highly similar to those that use biochemical analysis data [27]. The MFI index showed a similar dose-dependent tendency as the FLAV index after the second stage of treatment (Figure 2D).

### 2.2. Anthocyanins Content

**Figure 3:**
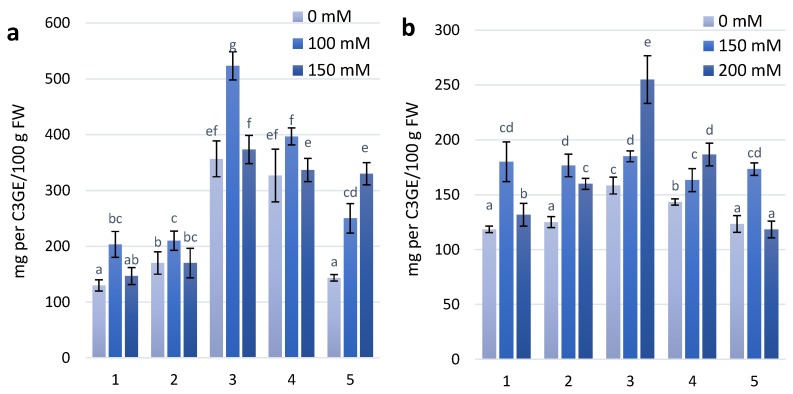
Anthocyanins content in wheat leaves under effect of different NaCl concentrations (**a**)—first stage of treatment; (**b**)—second stage of treatment. Wheat genotypes: 1—Citrus yellow, 2—KM 53-14 Blue, 3—KM 178-14 purple, 4—Skorpion Blue aleurone, 5—PS Karkulka purple. The columns represent the mean values ± S.E. for six replicates. Statistically significant differences among treatments at each time are indicated by different small letters (Duncan test, *p* < 0.05).

The experimental wheat genotypes have been characterized by different anthocyanin contents (Figure 3), which could affect the specific development of adaptive responses to increasing salinity stress [25].

Coloured plant genotypes that have high anthocyanin content are a good object to study abiotic tolerance, especially salt-tolerant traits. Two wheat genotypes, KM 178-14 purple and Skorpion Blue aleurone, had the highest anthocyanin content in the leaves among all experimental variants. At the first stage of the experimental treatment with 100 mM NaCl, increasing anthocyanin content was observed in almost all experimental variants. Under treatment with 150 mM NaCl, anthocyanin content was similar to the control level in all experimental variants, except for the wheat genotype PS Karkulka. The higher content of anthocyanins did not result in development of the same adaptation to salt stress in the genotypes. At the second stage of the experimental treatment, genotypes (KM 178-14 purple and Skorpion Blue aleurone) with higher anthocyanin content at 100 mM NaCl had no changes in anthocyanin content. The genotype KM 178-14 purple showed a significant increase in anthocyanin content of 30% at 200 mM NaCl compared to the control. Other genotypes (Citrus yellow, KM 53-14 Blue and PS Karkulka) have been characterized by an increase in anthocyanins at 150 mM NaCl, but at 200 mM NaCl, they were similar to the level of the control.

### 2.3. Proline Content

**Figure 4:**
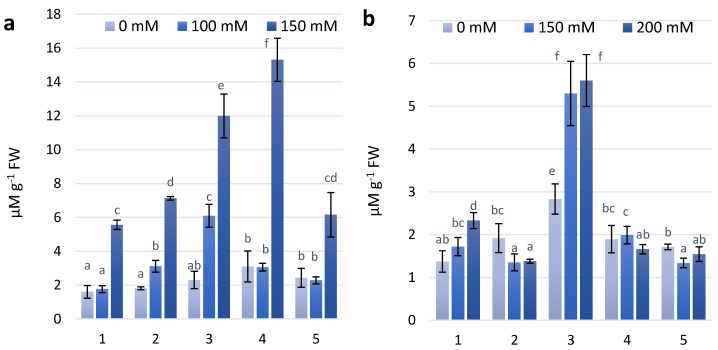
Proline content in wheat leaves under effect of different NaCl concentrations (**a**)—first stage of treatment; (**b**)—second stage of treatment. Wheat genotypes: 1—Citrus yellow, 2—KM 53-14 Blue, 3—KM 178-14 purple, 4—Skorpion Blue aleurone, 5—PS Karkulka purple. The columns represent the mean values ± S.E. for six replicates. Statistically significant differences among treatments at each time are indicated by different small letters (Duncan test, *p* < 0.05).

The results of the presented experimental work found that wheat genotypes characterized by increased anthocyanin content in the first and second stages of salt treatment show increasing proline content. In the first stage, a significant increase in proline content was observed for the wheat genotype KM 178-14 purple (Figure 4A). Under the second stage of treatment with 100 mM NaCl, a significant increase in proline content was observed for the KM 53-14 Blue and KM 178-14 purple wheat genotypes. However, under the 200 mM NaCl treatment in the second stage of treatment, all experimental genotypes showed increased proline content, especially the coloured cultivars KM 178-14 purple and Skorpion Blue aleurone (Figure 4B).

### 2.4. Lipid Peroxidation Assay

**Figure 5:**
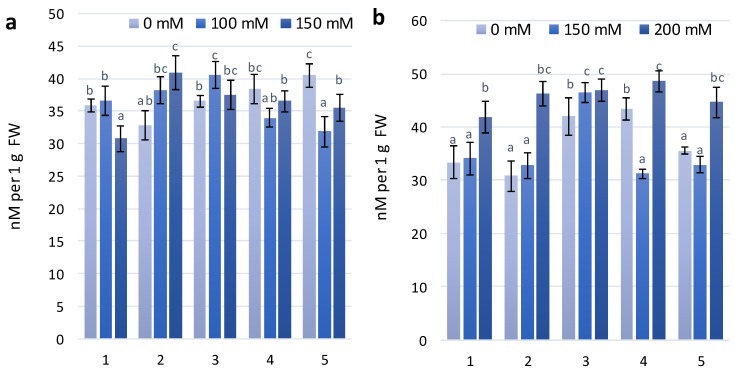
MDA content in wheat leaves under effect of different NaCl concentrations (**a**)—first stage of treatment; (**b**)—second stage of treatment. Wheat genotypes: 1—Citrus yellow, 2—KM 53-14 Blue, 3—KM 178-14 purple, 4—Skorpion Blue aleurone, 5—PS Karkulka purple. The columns represent the mean values ± S.E. for six replicates. Statistically significant differences among treatments at each time are indicated by different small letters (Duncan test, *p* < 0.05).

Lipid peroxidation (LP) is a biochemical marker for ROS-mediated damage. MDA is one of the better known secondary metabolite of LP [28,29]. The results of the experimental work consisting of two stages of NaCl treatment have shown the tendency to increase the MDA level in KM 53-14 Blue and KM 178-14 purple wheat genotypes, whereas other wheat genotypes were almost on a control level after the first treatment (Figure 5A). The significant increase of MDA accumulation was observed in the second treatment with concentration of 200 mM NaCl in all experimental variants (Figure 5B). Under the concentration 100 mM, almost all experimental variants have been characterized by MDA content equal to the control level, except of the genotypes Skorpion Blue aleurone and PS Karkula purple, in which we observed the decreasing tendency of MDA.

### 2.5. Plant Biomass

It has been suggested that stimulation of antioxidative responses under salt stress may result in comparable variability in growth sustainability [23]. An increase in the dry weight of roots under both concentrations of NaCl treatment was observed for the wheat genotype KM 178-14 purple. At a concentration of 200 mM NaCl, a higher dry weight was observed more than two times compared to the control for genotypes KM 178-14 purple, Skorpion blue aleurone, PS Karkulka (Table 1). These genotypes were also characterized by a high anthocyanin content in the control variants and different anthocyanin changes during the development of salt stress (Figure 2). The presence of anthocyanins as antioxidants can partly affect the development of stress adaptation responses under salt stress.

The dry weight of shoot biomass after salt stress influence increased again in Skorpion blue aleurone and PS Karkulka, but for genotype KM 178-14, the purple dry weight of shoots was on the control level. The genotype Citrus yellow, with a lower anthocyanin content, was characterized by a decrease in dry weight under both NaCl concentrations (150 mM and 200 mM). The genotype KM, 53-14 Blue demonstrated decreased shoot dry weight by 45% compared to the control after treatment with 200 mM NaCl.

### 2.6. Na^+^ and K^+^ Level

The content of Na^+^ and K^+^ obtained at different levels of salinity on the basis of DW was more than 3 times greater than the control, with a significant increase of both ions under salt stress. For some wheat genotypes, a significant increase in Na^+^ and K^+^ was observed under treatment with 150 mM NaCl (KM 53-14 Blue and KM 178-14 purple) and under treatment with 200 mM NaCl (Skorpion Blue aleurone and PS Karkulka). The Na^+^/K^+^ ratio was decreased in all experimental wheat genotypes (Table 2).

## 3. Discussion

In the present research, to discover the development of adaptive responses of different wheat genotypes under salt stress, non-destructive chlorophyll fluorescence techniques were used for the screening of biologically active compounds of a phenolic nature and some photosynthesis parameters. The majority of published vegetation indices for non-invasive remote sensing techniques are not responsive to fast changes in the status of plant photosynthesis under the effects of common environmental stressors [30]. The SFR index is connected with chlorophyll concentration in the leaves [27]. It was shown that under salt stress the SFR values at the first stage of treatment with a concentration of 100 mM in the two coloured genotypes, KM 178-14 purple and Skorpion Blue aleurone found no significant changes compared to the control. However, at the second stage of treatment, with increasing salt concentration, the SFR value decreased.

The level of photosynthetic pigments can be reduced under the effect of different stressful environments [30]. Plus the kinetics of pigments accumulation can be different at the two investigated developmental stages like 2-d-old and 10-d-old leaves [31] with differences between control variants at two investigated stages. The difference in salt tolerance may be connected with plant ontogenetic stages and plant species. Photosynthetic ability is significantly decreased in plants under salinity stress, which affects plant growth and development [32]. Decreasing photosynthetic ability by increased salt stress was connected to the inhibition of carbon uptake via specific metabolic processes, changes in photochemical capacity, lower stomatal conductance or a combination of these factors [33,34,35].

Flavonol accumulation, which is also a part of the adaptive reaction to salt stress, was observed after the first stage of treatment with NaCl (100 mM and 150 mM). After the second stage of treatment with 150 mM and 200 mM NaCl, we found a significant difference in the flavonol reaction between the coloured wheat genotypes (with higher anthocyanins content in grains) and non-coloured (with higher carotenoids content in grains). The FLAV and MFI indices have shown a similar dose-dependent tendency of flavonol content increase after the second stage of treatment in the coloured wheat genotypes. The grains of these wheat genotypes have been characterized by the presence of anthocyanins as well [36].

Intensive detoxification of reactive oxygen species (ROS) is a key factor in improving the tolerance of plants to salinity effects. An analysis of phenylpropanoid metabolism at the gene and enzyme levels showed that oxidative damage was lower when flavonols accumulated to a higher degree [37]. Therefore, the higher flavonol accumulation in coloured wheat genotypes (KM 178-14 purple and Skorpion Blue aleurone) showed faster development of adaptive responses to salt stress.

It was estimated that high contents of proline and anthocyanin support an active protective response to salinity [28]. At the first and second stages of salt treatment the coloured wheat genotypes characterized by higher anthocyanin content show increasing proline content. After first treatment the wheat genotypes KM 178-14 purple shows the highest increase in proline content. At the second stage of treatment with a concentration of 200 mM NaCl all experimental genotypes showed increased proline content.

Some studies have cited that the build-up of amino acid proline is an essential regulatory factor under salt stress and may be one of the mechanisms underlying their higher salt tolerance [38,39,40]. Matysik et al. (2002) showed that proline mitigates salt stress-induced increases in the carboxylase and oxygenase activities of Rubisco [41]. It protects plants from ROS damage by suppressing singlet oxygen. The early responses to salt and water stress have been found to be mostly equal [42]. Slama et al. (2015) found that in plants grown under salt stress, the elevation of the synthesis of proline can be regarded as an adaptation strategy in this species when exposed to salt stress [10]. Organic osmolytes can indirectly participate in osmotic adjustment by changing K^+^ flow across membranes, thus reducing sodium-induced efflux of such inorganic osmolytes [42].

Increased lipid peroxidation (LP) and proline contents were observed to significantly accumulate in barley and *Catharanthus roseus* seedlings under salinity effects [43,44]. LP process changes depend on plant sensitivity and the degree of influence stressor. The effect of increasing NaCl doses on the accumulation of proline and LP can result in their high correlation with the tolerance capacity of plant cultivars [45,46]. Low MDA values, as an important indicator of a high oxidative damage-limiting capacity, were shown to be important for tolerating the salinity [46,47].

The results of our experiment showed the different development of an LP process in different wheat genotypes. It was found that in salt-stressed seedlings, MDA contents were negatively correlated with the accumulation of proline and total anthocyanins [27,47]. The results of the experimental work on seedings have shown that Citrus yellow and KM 53-14 Blue cultivars were salt-stress sensitive genotypes compared to other tested varieties. Different changes in MDA, proline and anthocyanin accumulations under salt stress have been observed for coloured and non-coloured wheat genotypes. It can provide evidence for the development of adaptive responses under salt stress the wheat genotypes tested in the experiment.

The tolerant genotypes of wheat showed a better capacity to maintain the low accumulation of Na^+^, higher shoot K^+^ concentrations, stable osmotic potential, increased values of PSII activity, lower non-photochemical quenching (NPQ) and maximal photochemical efficiency derived in the significantly greater dry weight production detected under salt stress [25,26]. Low Na^+^ accumulation in leaf can be used as the best screening criteria, employing a large set of genotypes in a breeding program [48]. The dry weight of shoot biomass after salt stress influence increased again in Skorpion blue aleurone and PS Karkulka. The genotype Citrus yellow, with lower anthocyanin content, was characterized by a decrease in dry weight under both NaCl concentrations.

The content of Na^+^ and K^+^ obtained at different levels of salinity on the basis of DW was more than three times greater than the control, with a significant increase of both ions under salt stress. Cramer et al. (1985) demonstrated that in the presence of high NaCl concentrations, Na ^+^ moves Ca from the plasmalemma of root cells, resulting in increased membrane permeability that causes K efflux and alteration of the selectivity ratio K^+^/Na^+^ [49]. This suggests that plants that are successfully grown in saline conditions are those that maintain a higher K^+^/Na^+^ ratio in their cytoplasm than in the rhizosphere [50].

The toxicity of excessive salt on the cell level corresponds to the Na^+^ level accumulated in the cells [51]. In plants, K^+^ is engaged in cell metabolism, photosynthesis, and protein synthesis and is also essential for regulating enzyme activation and stomatal movement [52]. The regulation of Na^+^ uptake and support of the relative Na^+^/K^+^ ratio can be recognized as an important cellular mechanism that assists plant adaptation to saline environments [53].

Siddiqui et al. (2017) obtained similar results regarding Na^+^ ions. In the wheat leaves of 10 cultivars, significant increases in Na^+^ content, but decreases in K^+^ content were found under salinity stress conditions [54]. The ratio of Na^+^/K^+^ was significantly increased at all NaCl concentrations. The different plant genotypes reveal different salt tolerance mechanisms. These mechanisms are all based on the function and regulation of K^+^ and Na^+^ transporters and H^+^ pumps, which create the driving force for K^+^ and Na^+^ transport [55,56]. It can be concluded that the contribution of Na^+^ and K^+^/H^+^ antiporter proteins to plant salt stress tolerance is involved in K^+^ homeostasis support rather than Na^+^ sequestration into the vacuole.

The water content (WC) increased at high doses of NaCl under both treatment levels. During the growth phase and after salt application, the WC was effectively protected. This content is one of the key indicators of plant water status. Relative turgidity was less affected by stress and reflects good efficiency in saving water. This improvement can be explained, in part, by the effective accumulation of organic osmolytes, which suggests that the existence of mechanisms of osmotic adjustment lead to the protection of the structural and functional integrity of the tissue [10]. On the other hand, the improvement of the water status of plants may be, in part, a consequence of the lack of a significant reduction in stomatal conductance. It was found that salinity significantly reduced root hydraulic conductance [57]. In contrast, in cotton and common bean, WC decreased with salt stress at low rates of salt (50 mM) due to osmotic adjustment and a decrease in transpiration [58].

## 4. Materials and Methods

### 4.1. Plant Material

**Figure 6:**
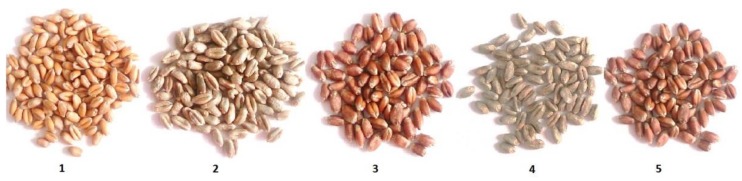
Coloured wheat seeds of the genotypes used in experiments 1—Citrus yellow, 2—KM 53-14 Blue, 3—KM 178-14 purple, 4—Skorpion Blue aleurone, 5—PS Karkulka purple.

Seeds of five wheat genotypes (*Triticum* sp.) with different pigments (Citrus yellow, KM 53-14 Blue, KM 178-14 purple, Skorpion Blue aleurone, and PS Karkulka purple) were provided by the Agricultural Research Institute Kromeriz, Kromeriz, Czech Republic, respectively. The characteristics of coloured grains were based on the visual pink, blue and yellow colour assessment (Figure 6).

Coloured grains have blue aleurone (Ba genes), purple pericarp (Pp genes) and yellow endosperm (Psy genes), which are determined by the presence of anthocyanins and carotenoids [59]. The blue aleurone (Ba genes) pericarp got grains of KM 53-14 Blue and Skorpion Blue aleurone wheat genotype. The purple pericarp (Pp genes) got grains of KM 178-14 purple and PS Karkulka purple wheat genotype. The yellow endosperm (Psy genes) have grains of Citrus yellow wheat genotype.

### 4.2. Growth Conditions

Sterile sandy soil of fine texture was filled in plastic pots (12 cm diameter) to conduct culture under controlled conditions. Grains were washed extensively with distilled water and sterilized with 5% sodium hypochlorite for 5 min. Then, seeds were sown in sandy soil directly watered with 3 mL of 1/4 strength Hoagland’s nutrient solution [60]. The germination process was under controlled conditions of 25 ± 2 °C. The controlled growing conditions in a growing chamber had the following parameters: relative humidity of 60–70% and light/dark regime of 16/8 h at 25/20 °C. Four pots per treatment with 6 plants per pot were harvested after 30 days of the experiment. Pots were irrigated every three days, alternatively with filtrated water and a nutrient solution, according to Etherton (1963) [61], until they were 4 weeks old. It was used different concentrations of salinity treatments (0 (Control), 100, 150 and 250 mM NaCl). The control plants only received nutrient solution. To avoid osmotic shock due to high concentrations, plants were started on lower concentrations 100 and 150 mM NaCl (first stage of treatment), then the concentration was increased after 5 days with 50 mM NaCl, until wheat genotypes reached the concentration 150 and 200 mM NaCl (second stage of treatment). Tap water was used daily to balance the water presence after evaporative loss in plant seedlings.

### 4.3. Growth Parameters

After harvest, the shoot fresh weight (FW) was measured. The dry weight (DW) was estimated after putting plant samples in a forced-draft oven at 60 °C for 48 h or until a fixed weight was obtained.

Water content (g H_2_O g^−1^ DW) = (FW − DW)/DW

### 4.4. Chlorophyll Fluorescence Records and Analyses Using the Fluorescence Excitation Ratio Method

Chlorophyll fluorescence analysis was performed using the portable optical fluorescence sensor Multiplex-3^®^ (Force-A, Paris, France). Multiplex-3^®^ is a multi-parametric, hand-operated sensor. The determination principle is based on light-emitting-diode excitation and a filtered photodiode. The portable optical fluorescence sensor Multiplex-3^®^ is arranged to work in the laboratory, greenhouse and field conditions. The Multiplex-3^®^ sensor has three red-blue-green LED matrices emitting light at 153 and 635 nm (red), 470 nm (blue) and 516 nm (green). There are three integrated photodiode detectors for fluorescence recording: far-red, red and yellow [29]. The values of fluorescence measured at UV (375 nm), green light (516 nm), red light (635 nm) and far-red light (735 nm) have been used.

The evaluation of phenolic compound contents in plants was performed via the calculation of fluorescence values detected after excitation by light of the defined wavelengths (details are below). Similar to the spectrophotometric method for assessing leaf absorbance, the parameters were based on Beer-Lambert’s law and calculated as the logarithm of the fluorescence ratio values.

The UV absorbing compound (mostly flavonol) content described by the flavonoid (FLAV) index [62] was estimated using the modified formula [27] as the logarithm of the ratio of the red-light induced far-red fluorescence (FRF_R_) and the UV-induced far-red fluorescence (FRF_UV_):

FLAV = log[FRF_R_/(k_UV_ × FRF_UV_)]

Similarly, the ANTH Index that provides estimates of green-light absorbing components (logFER_R/G_), mostly red-coloured anthocyanins and flavonoids, was determined as the logarithm of the ratio of the red-light induced fluorescence (FRF_R_) and the green light-induced fluorescence (FRF_G_):

ANTH = log[FRF_R_/(k_G_ × FRF_G_)]

The correction coefficients k_UV_ or k_G_ were applied to measurements of fluorescence to avoid negative values [27]. The constant values of the coefficients were used as the minimum values of the FRF_UV_/FRF_R_ and FRF_G_/FRF_R_ ratios found in the database that contains several thousand records from over three hundred plant species grown in diverse environments. The same constants have been used when processing data across all experiments and cultivars. We also calculated the modified flavonoid index (MFI), which provides a better estimate of total flavonoid content when plants with different colours are compared [27]. The logarithm of the ratio of the red-light induced fluorescence (FRF_R_) and the green light-induced fluorescence (FRF_G_) was used to calculate the MFI parameter.

MFI = log[2 × FRF_R_/(kG × FRF_G_ + k_UV_ × FRFU_V_)]

The values of correction coefficients (k_G_, k_UV_) for MFI were the same as for ANTH and FLAV.

Chlorophyll content was estimated from values of fluorescence measured at 735 nm (FRF) and 685 nm (RF) after excitation by red light (635 nm). The simple fluorescence ratio (SFR) was calculated:

SFR = FRF_R_/RF_R_

Because the diameter of the measuring area was only 50 mm, 6–7 measurements were taken on each plant in different positions to account for heterogeneity in leaf colour and structure. This number of measurements from the top view provides sufficient data to characterize the entire plant. The number of measurements for each plant was 30.

### 4.5. Proline Assay

The extraction was made by the method of Troll and Lindsley (1955) and simplified based on Wittmer (1987) [63,64]. One hundred milligrams of fresh material was placed with 2 mL of 40% methanol. The reaction mixture was heated at 85 °C in a water bath for 1 h. After cooling, 1 mL was removed from the extract to which 1 mL of acetic acid and 1 mL of the mixture containing 120 mL distilled water + 300 mL of ortho-phosphoric acid was added. The resulting solution was boiled for 30 min. After cooling, 5 mL of toluene was added. Two phases were separated and the upper phase (organic phase) was recovered. Absorbance measurements were determined using a Jenway UV/Vis. 6405 spectrophotometer (Jenway, Chelmsford, UK) at 528 nm. Proline concentration was estimated from a standard curve.

### 4.6. Lipid Peroxidation (LP) Assay

LP in leaves was identified by estimating the malondialdehyde (MDA) concentration [65]. Plant material (0.2 g) was homogenized in 3 mL of 0.1 mol kg^−1^ Tris buffer containing 0.3 mol kg^−1^ NaCl. Two millilitres of 20% trichloroacetic acid (TCA) containing 0.5% thiobarbituric acid and 2 mL of 20% TCA were added. The mixture was heated at 95 °C for 30 min. Then, the homogenate was centrifuged at 10,000× *g* for 5 min. The absorbance of the supernatant was measured at 532 nm with use of spectrophotometer Jenway UV/Vis. 6405 (Jenway, Chelmsford, UK).

### 4.7. Anthocyanin Estimation

Plant material (0.1 g) was soaked in 3 mL of acidified methanol (1% *v*/*v* HCl) for 12 h in darkness at 4 °C with occasional shaking. The mixture was centrifuged for 10 min at 14 000 rpm at 4 °C. Absorption of the extracts was estimated spectrophotometrically at 530 and 657 nm wavelengths on spectrophotometer Jenway UV/Vis. 6405 (Jenway, Chelmsford, UK). The blank was acidified methanol. The anthocyanin concentration was revealed as mg·g^−1^ DW and was calculated by the following formula:

Anthocyanins = [OD530 − 0.25 OD657] × TV/[d wt × 1000](1)

OD = optical density; TV = total volume of the extract (mL); d wt = weight of the dry leaf tissue (g).

### 4.8. Na^+^ and K^+^ Accumulation

Measurements of Na^+^ and K^+^ accumulation in the flag leaf were carried out 3 days after flowering. Oven-dried simple flag leaves were finely ground before passing through a 2-mm sieve. Samples (0.5 g) were mixed with 10 mL of concentrated nitric acid and 3 mL of perchlorate acid in digesting tubes for 12 h and then dissolved at 300 °C for 6 h. The concentration of K^+^ and Na^+^ was detected using an atomic absorption spectrophotometer (Sherwood 410, Cambridge, UK).

### 4.9. Statistical Analysis

Statistical analyses were performed using two factor (genotype x salt concentration) analysis of variance (ANOVA) and Duncan’s multiple range test performed at *p* = 0.05 (STATISTICA 10, StatSoft, Tulsa, OK, USA). Two terms of measurements were analysed separately. Mean values were calculated from six plants per cultivar in each experimental variant. Data are presented as mean ± standard error from six replicates (SE).

## 5. Conclusions

In the presented work has been presented the role of anthocyanins in the development salt tolerant stress responses in wheat plants. The coloured wheat genotypes, which were used as models to study the role of anthocyanins in the development of adaptive response to salt stress, may be used in the farther agricultural cultivation practices. It was found that higher flavonol and anthocyanin accumulation in coloured wheat genotypes showed a faster development of adaptive responses of wheat genotypes to salt stress. Furthermore, the KM 178-14 purple, Skorpion blue aleurone, and PS Karkulka, genotypes demonstrated an increased content of anthocyanins, proline and flavonol in the presence of NaCl and are more salt tolerant than Citrus yellow and KM 53-14 Blue, as most of the parameters (especially growth) were rarely influenced or affected by the use of 150 mM NaCl.

## Figures and Tables

**Table 1:** Fresh and dry mass after salt stress influence.

**Wheat genotypes**	**Fresh Weight ^1^**			**Dry Weight ^1^**		
	**Roots**					
	**Control**	**150 mM**	**200 mM**	**Control**	**150 mM**	**200 mM**
Citrus Yellow	0.048 ± 0.019 ^ab^	0.069+0.023 ^a^	0.23 ± 0.07 ^b^	0.028 ± 0.009 ^a^	0.019 ± 0.003 ^ab^	0.017 ± 0.009 ^b^
KM 53-14 Blue	0.056 ± 0.003 ^b^	0.042 ± 0.004 ^a^	0.086 ± 0.02 ^c^	0.011 ± 0.005 ^a^	0.015 ± 0.002 ^a^	0.016 ± 0.008 ^a^
KM 178-14 Purple	0.042 ± 0.001 ^a^	0.050 ± 0.016 ^a^	0.13 ± 0.05 ^b^	0.006 ± 0.001 ^a^	0.010 ± 0.004 ^a^	0.013 ± 0.006 ^a^
Skorpion Blue Aleur.	0.091 ± 0.022 ^a^	0.038 ± 0.014 ^b^	0.079 ± 0.025 ^a^	0.007 ± 0.003 ^a^	0.007 ± 0.001 ^a^	0.020 ± 0.012 ^a^
PS Karkulka	0.046 ± 0.012 ^b^	0.039 ± 0.014 ^b^	0.228 ± 0.091 ^a^	0.016 ± 0.006 ^ab^	0.011 ± 0.002 ^b^	0.023 ± 0.007 ^a^
	**Shoots**					
	**Control**	**150 mM**	**200 mM**	**Control**	**150 mM**	**200 mM**
Citrus yellow	0.278 ± 0.076 ^a^	0.297 ± 0.085 ^a^	0.233 ± 0.073 ^a^	0.038 ± 0.011 ^ab^	0.043 ± 0.014 ^a^	0.019 ± 0.008 ^b^
KM 53-14 Blue	0.194 ± 0.117 ^ab^	0.187 ± 0.028 ^a^	0.086 ± 0.032 ^b^	0.028 ± 0.014 ^a^	0.028 ± 0.024 ^a^	0.018 ± 0.004 ^a^
KM 178-14 Purple	0.252 ± 0.062 ^a^	0.188 ± 0.06 ^a^	0.127 ± 0.047 ^b^	0.032 ± 0.009 ^a^	0.032 ± 0.012 ^ab^	0.015 ± 0.007 ^b^
Skorpion Blue Aleur.	0.556 ± 0.085 ^a^	0.283 ± 0.071 ^b^	0.079 ± 0.035 ^c^	0.025 ± 0.009^a^	0.042 ± 0.009 ^a^	0.074 ± 0.013 ^b^
PS Karkulka	0.209 ± 0.069 ^a^	0.241 ± 0.063 ^a^	0.228 ± 0.052 ^a^	0.030 ± 0.011 ^a^	0.042 ± 0.009 ^a^	0.046 ± 0.015 ^a^

^1^ Values are mean ± S.E. for six replicates. Statistically significant differences among treatments at each time within one genotype are indicated by different small letters (Duncan test; *p* < 0.05).

**Table 2:** Effects of different levels of salinity on total dry weight, shoot water content after 30 days of sowing of investigated wheat cultivars.

Wheat Cultivars ^1^	Salinity Level (mM NaCl)	Water Content (g H_2_O g^−^^1^ DW)	Total Dry Weight (g/plant)	Na^+^ mmol/g DW	K^+^ mmol/g DW	K^+^/Na^+^ Ratio
Citrus Yellow	0 mM	6.30 ± 0.650 ^b^	0.066 ± 0.016 ^a^	0.692 ± 0.007 ^b^	0.179 ± 0.002 ^b^	0.259
	150 mM	5.99 ± 0.588 ^b^	0.062 ± 0.017 ^a^	1.602 ± 0.097 ^a^	0.333 ± 0.020 ^a^	0.208
	200 mM	12.15 ± 5.24 ^a^	0.036 ± 0.017 ^a^	1.663 ± 0.129 ^a^	0.345 ± 0.027 ^a^	0.208
KM 53-14 Blue	0 mM	5.96 ± 1.50 ^a^	0.039 ± 0.019 ^a^	0.918 ± 0.085 ^b^	0.238 ± 0.022 ^b^	0.259
	150 mM	6.92 ± 0.341 ^a^	0.043 ± 0.016 ^a^	2.582 ± 0.080 ^a^	0.508 ± 0.016 ^a^	0.197
	200 mM	6.47 ± 5.04 ^a^	0.034 ± 0.012 ^a^	2.272 ± 0.162 ^a^	0.447 ± 0.032 ^a^	0.197
KM 178-14 Purple	0 mM	6.86 ± 0.91 ^b^	0.038 ± 0.010 ^a^	0.643 ± 0.054 ^c^	0.148 ± 0.012 ^c^	0.231
	150 mM	4.65 ± 1.66 ^b^	0.042 ± 0.011 ^a^	2.606 ± 0.138 ^a^	0.455 ± 0.024 ^a^	0.175
	200 mM	9.92 ± 2.79 ^a^	0.056 ± 0.009 ^a^	1.597 ± 0.093 ^b^	0.295 ± 0.017 ^b^	0.185
Skorpion Blue Aleurone	0 mM	22.11 ± 5.88 ^a^	0.027 ± 0.012 ^b^	0.664 ± 0.074 ^b^	0.153 ± 0.017 ^b^	0.185
	150 mM	5.63 ± 0.38 ^c^	0.049 ± 0.010 ^b^	1.636 ± 0.093 ^a^	0.302 ± 0.017 ^a^	0.185
	200 mM	3.98 ± 0.93 ^b^	0.094 ± 0.025 ^a^	1.791 ± 0.090 ^a^	0.331 ± 0.017 ^a^	0.231
PS Karkulka	0 mM	6.62 ± 1.73 ^b^	0.046 ± 0.017 ^a^	0.633 ± 0.023 ^c^	0.146 ± 0.005 ^b^	0.231
	150 mM	4.69 ± 0.46 ^b^	0.053 ± 0.011 ^a^	1.634 ± 0.093 ^b^	0.301 ± 0.017 ^a^	0.185
	200 mM	10.10 ± 3.67 ^a^	0.069 ± 0.020 ^a^	1.972 ± 0.063 ^a^	0.345 ± 0.011 ^a^	0.175

^1^ Values are mean ± S.E. for six replicates. Statistically significant differences among treatments at each time within one genotype are indicated by different small letters (*p* < 0.05).
